# Outcomes and rate of return to play in elite athletes following arthroscopic surgery of the hip

**DOI:** 10.1007/s00264-021-05077-3

**Published:** 2021-06-19

**Authors:** R. Elwood, O. El-Hakeem, Y. Singh, H. Shoman, O. Weiss, V. Khanduja

**Affiliations:** 1 School of Clinical Medicine, University of Cambridge, Cambridge, UK; 2 Addenbrooke’s - Cambridge University Hospital NHS Foundation Trust, Hills Road, Cambridge, CB2 0QQ UK; 3 Young Adult Hip Service, Department of Trauma and Orthopaedics, Addenbrooke’s - Cambridge University Hospitals NHS Foundation Trust, Box 37, Hills Road, CB2 0QQ Cambridge, UK; 4 Mobius Health, Nuffield Hospital, 4 Trumpington Road, Cambridge, CB2 8AF UK

**Keywords:** Hip arthroscopy, Elite, Athletes, Professional sports, Outcomes, Return to play

## Abstract

**Background:**

The tremendous physical demands of elite performance increase the risk of elite athletes sustaining various orthopaedic injuries. Hip pain is common in high-level athletes representing up to 6% of all athletic injuries. Expedient diagnosis and effective treatment are paramount for their future sporting careers and to prevent subsequent joint degeneration.

**Purpose:**

This systematic review aimed to evaluate the outcome and the rate of return to play (RTP) following arthroscopic procedures in the hip (osteoplasty, chondroplasty, labral repair and/or debridement, capsulotomy, capsulorrhaphy or any soft tissue procedure) in elite athletes. Elite athletes were defined as those who represented their country in international contests or were competing professionally for the purpose of this study.

**Methods:**

A computer-based systematic search, following the PRISMA Guidelines, was performed on CENTRAL, PUBMED, EMBASE, SCOPUS, EBSCO, Google Scholar and Web of Science from inception until January 1, 2020, identifying studies that looked at return to sports post-hip arthroscopy in elite athletes. Weighted means were calculated for the RTP rate and duration and for patient-reported outcome measures (PROMs).

**Results:**

After eligibility screening, 22 articles were included with a total of 999 male and seven female patients, 1146 hips and a mean age of 28.4 ± 3.2 years. The mean follow-up period was 35.8 ± 13.4 months and 15.9 ± 9.6% of athletes had undergone bilateral procedures. Overall, 93.9% (95% CI: 90.5, 96.6, *P* < 0.0001) of patients demonstrated RTP after 6.8 ± 2.1 months post-surgery and all PROMs improved post-operatively. During follow-up, 9.6% (95% CI: 5.2, 15.2, *P* = 0.025) patients needed further intervention.

**Conclusion:**

A high percentage of elite athletes return to the same level of competition after hip arthroscopy, with a low rate of further interventions. Hip arthroscopy appears to be an efficacious treatment for hip and/or groin pain, caused by pathologies such as FAI or labral tears, in elite athletes in the shorter term. Long term outcomes need further evaluation.

## Introduction

The physical demands associated with elite levels of play place elite athletes at an increased risk of sustaining a variety of orthopaedic injuries [11, 24, 28, 44]. Pain around the hip is common in high-level athletes and can be due to a variety of conditions and pathologies representing up to 6% of all athletic injuries [4, 12, 32, 48].

Elite athletes, representing their country in international contests or competing professionally, often rely on high-impact activities for high-level performance, such as jumping, sprinting and cutting [7]. Forceful hip flexion, abduction and external rotation combined with repetitive loading have been shown to promote chondrolabral dysfunction, separation between the labrum and articular margin [42] which may lead to further damage to the hip, development of early-onset hip osteoarthritis [1, 2, 6, 19, 20, 54] and eventually the need for total hip arthroplasty . Two of the most common hip pathologies among elite athletes are labral tears occuring acutely and femoroacetabular impingement (FAI) which in itself may lead to labral pathology. The latter results from abnormal contact between the proximal femur and acetabulum secondary to a loss of sphericity at the femoral head-neck junction (cam) or acetabular overcoverage of the femoral head (pincer). Furthermore, studies have shown that high-intensity sports during adolescence may also lead to the development of a cam-type deformity [34, 39, 49].

Such hip pathologies are a potential cause of significant disability and may substantially affect the subsequent performance and career longevity of elite athletes. As such, it is important that players, team physicians, athletic trainers and coaches understand the potential career risks and benefits when operative management is indicated.

Expedient diagnosis and effective treatment are paramount for allowing athletes to continue their sports career and prevent subsequent joint degeneration. The understanding and diagnosis of femoroacetabular impingement (FAI) has increased over recent years, and a number of these athletes were likely misdiagnosed as having a “strain” when the aetiology of pain was truly FAI [11, 18].

Recent studies have demonstrated high return to play (RTP) rates within an athletic population [31, 37]; however, no study has systematically looked at rates of RTP within an elite athletic population or reviewed the time to RTP, which is another important factor to consider for the elite athlete whose income may depend on their ability to compete. Several studies have shown the outcome of arthroscopic hip procedures to treat various hip pathologies among athletes in different sports professions. These prior studies demonstrated high rates of RTP in professional sports such as ice hockey, football, soccer, baseball, golf and basketball [14, 22, 26, 27, 29, 35, 43]. The performance outcomes and return to activity rates of elite athletes after hip arthroscopy are, however, currently unknown.

The purpose of this systematic review is to evaluate the outcome and the rate of return to play after arthroscopic procedures in the hip (osteoplasty, chondroplasty, labral repair and/or debridement, capsulotomy, capsulorrhaphy or any soft tissue procedure) amongst elite athletes. Our hypothesis is that there will be a higher level of return to play within the elite compared to the general athletic population, given the strong personal motivation, often financial, of these athletes and their access to quality resources and rehabilitation regimes.

## Methods

### Search strategy

A computer-based systematic search that followed the Preferred Reporting Items of Systematic Reviews and Meta-analysis (PRISMA) Guidelines [53] was performed using the following databases: CENTRAL (Cochrane Central Register of Controlled Trials), PUBMED, EMBASE, SCOPUS, EBSCO, Google Scholar and Web of Science Core Collection, for literature describing outcomes of hip arthroscopy among elite athletes.

All published studies from inception until January 1, 2020, were included in the systematic search. An *a priori* search algorithm using PubMed Medical Subject Headings (MeSH) terms was constructed. Duplicates were then removed electronically and manually. A detailed search strategy is described in the Appendix. The search function consisted of 3 search categories: studied population, procedure and confined to the hip joint. A total of seven combinations of keywords were combined together using Boolean terms AND/OR to create the following search strategy: “Athlete*” OR “Sport*” OR “Players” AND “Hip” AND “Arthroscopy” OR “Arthroscopic” OR “keyhole”.

First, a blinded and independent process of selection based on title and abstract was made by two authors. Secondly, all studies were then assessed for eligibility using the pre-defined inclusion and exclusion criteria by titles and abstracts until full-text review. When discrepancies were found between the authors, a third author gave the final input until a consensus was reached. Reference lists of included articles were also screened for relevant articles.

The protocol of this systematic review was registered and published in the international prospective register of systematic reviews (PROSPERO) under the registration number: CRD42018115004 (https://www.crd.york.ac.uk/prospero/).

### Eligibility criteria

All published studies, from inception until January 1, 2020, which reported the outcomes of hip arthroscopic surgery among elite athletes and met the following eligibility criteria, were included in the systematic search.

Studies were deemed eligible if they were written in English language and reported on human subjects with symptomatic hip pathology who have undergone primary hip arthroscopic (keyhole) surgery, including any of the following techniques: osteochondroplasty, chondroplasty, labral repair, reconstruction or labral debridement, capsulotomy or capsulorrhaphy and any soft tissue debridement. The subjects were required to be elite athletes of any sports, who have represented their country in international contests or were competing professionally. Minimum level IV evidence studies using Oxford Centre for Evidence-Based Medicine 2011 Levels of Evidence [38] were included. Exclusion of studies occurred if the subjects had a procedure using any implant, had any documented congenital or developmental paediatric hip disorders, such as Perthes disease, developmental dysplasia or slipped capital femoral epiphysis (SCFE) or had undergone any additional technique/procedure except the following: osteoplasty, chondroplasty labral repair and/or labral debridement, capsulotomy or capsulorrhaphy and any soft tissue debridement. Non-English language publications, animal or cadaveric studies, as well as reviews, hypothesis, technique, meta-analysis articles or oral presentations, were also excluded. Any excluded study, together with the reasons of exclusion, were noted.

### Quality assurance

All eligible studies, as determined by the inclusion/exclusion criteria, were assessed and measured using the Risk Of Bias In Non-randomized Studies of Interventions (ROBINS-I) scoring system and the Methodological Index for Non-Randomized Studies (MINORS) scoring. Included studies were rated by two independent reviewers, who were blinded to author, affiliations and publishing journal. Any disagreements between reviewers were discussed in a consensus meeting and an independent arbitrator was employed when consensus could not be met. The results of this are summarized in Table 1.

**Table 1:** Quality assurance of included studies

Author	Year	MINORS score (risk of bias)	ROBINS-I score
Jack et al. [16]	2020	High risk	Moderate
Jack et al. [17]	2019	High risk	Moderate
Sochacki et al. [51]	2019	High risk	Moderate
Sochacki et al. [50]	2019	High risk	Moderate
Lubbe et al. [23]	2018	High risk	Moderate
Barastegui et al. [5]	2018	High risk	Serious
Begly et al. [7]	2018	High risk	Moderate
Frangiamore et al. [13]	2018	High risk	Moderate
Locks et al. [22]	2018	High risk	Moderate
Nwachukwu et al. [36]	2018	High risk	Moderate
Schallmo et al. [46]	2018	Low risk	Low
Menge et al. [29]	2017	High risk	Moderate
Menge et al. [30]	2016	High risk	Moderate
Newman et al. [35]	2016	High risk	Moderate
Amenabar et al. [3]	2013	High risk	Moderate
Boykin et al. [9]	2013	High risk	Moderate
McDonald et al. [27]	2013	Low risk	Low
Hammoud et al. [15]	2012	High risk	Serious
Philippon et al. [43]	2010	High risk	Low
Philippon et al. [41]	2009	High risk	Low
Philippon et al. [40]	2007	High risk	Moderate
Saw et al. [45]	2004	High risk	Low

### Data extraction and data synthesis

The primary investigator (RE) extracted the relevant study data from the final pool of included articles and recorded this data on an Excel spreadsheet designed a priori.

Participant-specific demographics extracted from each study included the number of hips, gender distribution, mean age with range (years), mean BMI, length of follow-up, sports type, level of competition, surgical technique (labral repair or labral debridement) and rehabilitation intensity (duration and frequency). Outcome data, as presented in last follow-up, included clinical assessment (clinical scoring, pain scoring and level of satisfaction) and functional assessment, which included (rate to return to sports at the same competitive level, time to return to play, post-operative career lengths and retirement rates at the end of the follow-up).

### Statistical analysis

The method of data extraction and computation followed the approach outlined by the Cochrane Handbook for Systematic Reviews of Interventions.

The aggregate data of clinical studies were analyzed with a random effect proportion meta-analysis (chi-squared test—*χ*^2^), weighted for individual study size, with MedCalc (MedCalc Statistical Software version 18.9.1 (MedCalc Software bvba, Ostend, Belgium; http://www.medcalc.org; 2018)). According to all included studies, the alpha level was set at 0.05 and all p values were two tailed.

Weighted means were calculated for age, follow-up period, incidence of bilateral procedures, patient-reported outcome measures, rehabilitation regimes, rates of further interventions and career prognostic data (rate & time of return to play, career length and retirement rate) and were determined by study enrollment data. These outcomes were summarized in forest plots, which included studies that reported the necessary data for inclusion.

## Results

**Fig. 1:**
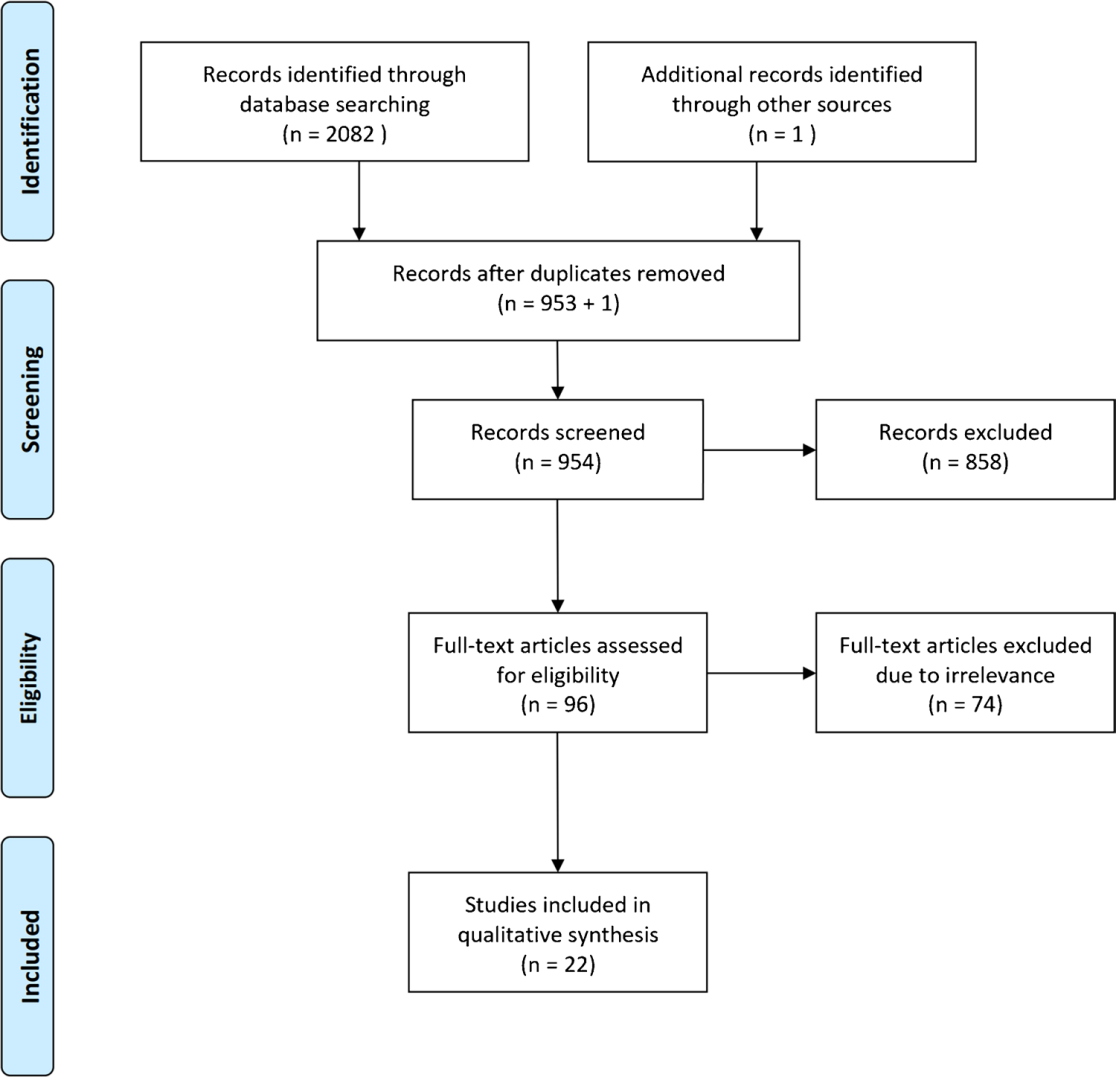
PRISMA flowchart

The search returned a total of 2082 articles through database searching and 906 duplicate results were removed. One article was identified through manual searches using cross referencing. After duplicates were removed, 954 articles were screened based of their titles and abstracts. The 96 full-text articles were screened for eligibility criteria, and 74 articles were excluded: one non-English language study, three-level V evidence articles, two papers with cohorts duplicated in one of our included articles, three review article, three abstracts, six papers did not report return to play outcomes and 56 papers were irrelevant. Overall, twenty-two studies looking at hip arthroscopic surgical outcomes among elite athletes met the eligibility criteria and were used in the final systematic review (Fig. 1).

### Patient demographics

In total, 999 male and seven female patients were identified, with 1146 hips and an adjusted mean age of 28.4 ± 3.2 years. The mean follow-up period, reported in five of the papers, was 35.8 ± 13.4 months. BMI data was lacking in most of the papers. The proportion of athletes who had undergone bilateral hip arthroscopies was 15.9 ± 9.6% (Table 2). The results are certainly skewed towards the male population, but unfortunately, this is what has been reported and available in the literature.

**Table 2:** Included studies examining outcomes of hip arthroscopy in elite athletes

			Enrolment				
Author	Year	Location	Patients (% of total cohort)	Hips (% of total cohort)	Male (%)	Mean age (y)	Sports type
Jack et al. [16]	2020	USA	23 (2.3%)	24 (2.1%)	100%	27.5	Basketball
Jack et al. [17]	2019	USA	50 (5.0%)	57 (5.0%)	100%	30.4	Baseball
Sochacki et al. [51]	2019	USA	71 (7.1%)	77 (6.7%)	100%	29.4	Ice hockey
Sochacki et al. [50]	2019	USA	55 (5.5%)	63 (5.5%)	100%	27.5	American football
Lubbe et al. [23]	2018	USA	64 (6.4%)	64 (5.6%)	100%	30.3	Ice hockey
Barastegui et al. [5]	2018	Spain	21 (2.1%)	21 (1.8%)	100%	26.5	Soccer
Begly et al. [7]	2018	USA	18 (1.8%)	24 (2.1%)	77.7%	25.6	Basketball
Frangiamore et al. [13]	2018	USA	44 (4.4%)	51 (4.5%)	100%	26.6	Baseball
Locks et al. [22]	2018	USA	24 (2.4%)	26 (2.3%)	100%	25	Soccer
Nwachukwu et al. [36]	2018	USA	40 (4.0%)	48 (4.2%)	100%	25.6	American football
Schallmo et al. [46]	2018	USA	180 (17.9%)	227 (19.8%)	100%	28.9	Mixed
Menge et al. [29]	2017	USA	51 (5.1%)	60 (5.2%)	100%	27	American football
Menge et al. [30]	2016	USA	60 (6.0%)	69 (6.0%)	100%	27	Ice hockey
Newman et al. [35]	2016	USA	20 (2.0%)	27 (2.4%)	100%	38	Golf
Amenabar et al. [3]	2013	Australia	26 (2.6%)	34 (3.0%)	100%	21.8	Australian Rules football
Boykin, et al. [9]	2013	USA	21 (2.1%)	23 (2.0%)	100%	28	Mixed
McDonald et al. [27]	2013	USA	120 (12.0%)	133 (11.6%)	100%	29.1	Mixed
Hammoud et al. [15]	2012	USA	38 (3.8%)	38 (3.3%)	100%	31	Mixed
Philippon et al. [43]	2010	USA	28 (2.8%)	28 (2.4%)	100%	27	Ice hockey
Philippon et al. [41]	2009	USA	1 (0.1%)	1 (0.09%)	100%	25	American football
Philippon et al. [40]	2007	USA	45 (4.5%)	45 (3.9%)	93.3%	31	Mixed
Saw et al. [45]	2004	UK	6 (0.6%)	6 (0.5%)	100%	–	Soccer

### Sports and level of competition

Four papers solely considered footballers, three were on soccer players, four were on (ice) hockey players, two were on baseball players, two looked at basketball players, one paper was on golfers and one paper was on Australian Rules footballers. The five remaining papers had a mixed cohort of athletes. The details of the sporting distribution of each paper are included in Table 2.

Three papers included professional athletes at the level of second national league or lower, 17 papers included professional athletes in the highest national league, three papers included players who had represented their country at an international level and one paper included some Olympic-level athletes. However, there may be some overlap between professional leagues and representation at the international or Olympic level, which may not have been reported. Four papers were not specific on the level of competition of the professional athletes that they included. Ice hockey, American football, baseball and soccer were the most reported sports, respectively (Table 3).

**Table 3:** Types of sports played by the study participants

Sport	Number of participants (%)	Number of studies (%)
Ice hockey	368 (36.6%)	9 (40.9%)
American football	248 (24.7%)	9 (40.9%)
Baseball	172 (17.1%)	7 (31.8%)
Soccer	72 (7.2%)	7 (31.8%)
Basketball	68 (6.8%)	5 (22.7%)
Golf	39 (3.9%)	3 (13.6%)
Australian rules football	26 (2.6%)	1 (4.5%)
Tennis	5 (0.5%)	2 (9.1%)
Skiing	2 (0.2%)	1 (4.5%)
Ice skating	2 (0.2%)	2 (9.1%)
Dance	2 (0.2%)	2 (9.1%)
Martial arts	1 (0.1%)	1 (4.5%)
Jockey	1 (0.1%)	1 (4.5%)

### Treatment (surgical technique, intra-operative findings/pathologies and post-op rehabilitation)

A total of 68.7% (691 patients) had been diagnosed with FAI, and 41.3% (415 patients) diagnosed with labral pathology. Rim lesions and chondral lesions were also common with 4.8% (48) and 11.3% (114) patients respectively across the cohort. Ligamentum teres injuries were reported for 6.9% (69) patients. One paper reported 3.5% (35) patients with micro-instability and another paper reported 2.7% (27) athletes with athletica pubalgia.

A variety of surgical techniques and procedures were performed across the papers. Osteochondroplasty was reported in 11 papers, with an average of 53.7% of patients across the eight papers that gave quantifiable data. Labral reconstruction or repair was reported in 15 papers with an average of 78.0% of patients undergoing the technique. Ten papers reported patients undergoing labral resection or debridement, performed on an average of 20.9% patients across these papers. Microfracture was reported in 14 papers, with an average of 18.2% patients across 13 papers. Acetabular rim trimming was reported in six papers, with an average of 75.2% patients undergoing the technique.

A consistently reported rehabilitation measure in the studies was weight-bearing protocols, which was reported in 14 studies. Twelve of these studies gave quantifiable data, with an average time of limited weight-bearing of 24.2 ± 10.2 days, including the patients that underwent longer rehabilitation after microfracture. The duration of limited weight-bearing protocols varies, but there is a consensus for eight weeks of limited weight-bearing following a microfracture. Six of these studies had a rehab regime where athletes were restricted to limited weight-bearing for two weeks or eight weeks if they had had microfracture.

### Patient-reported outcome measures (PROMs)

**Fig. 2:**
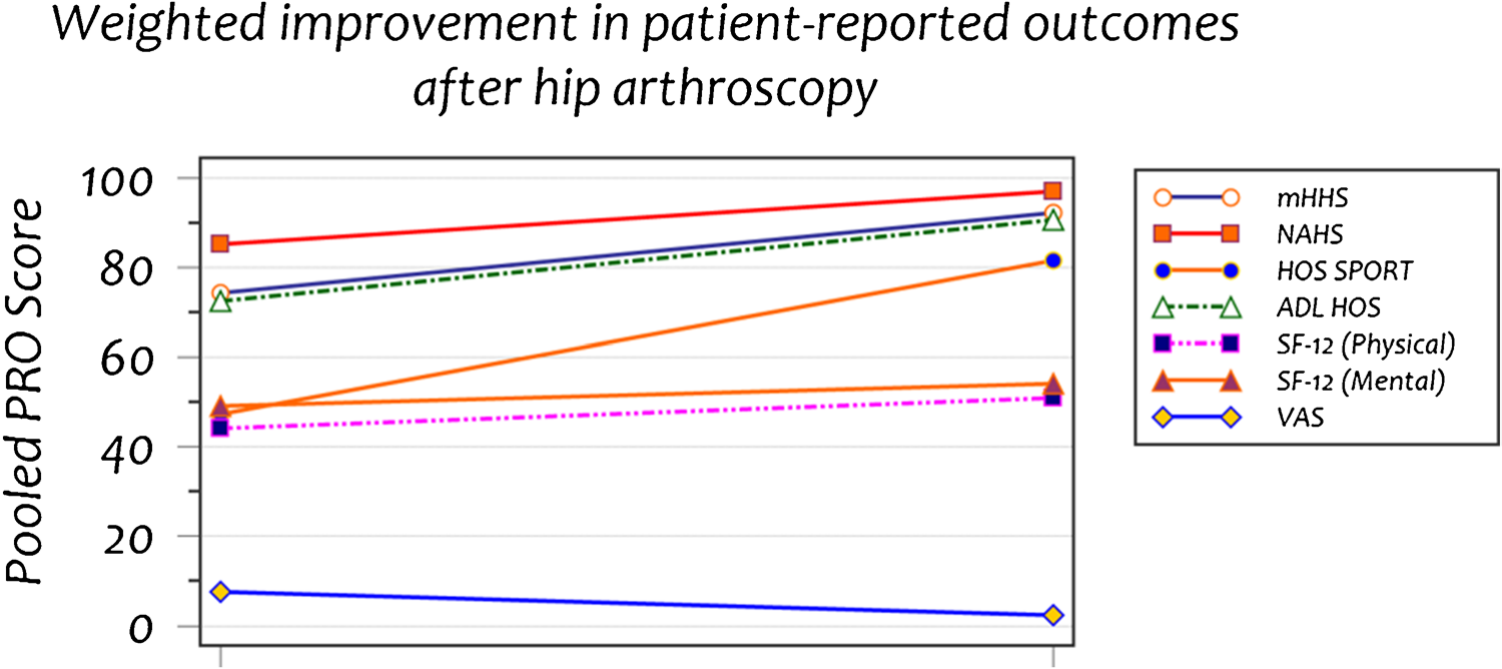
Patient-reported outcome measures of the included studies

Only 18.1% of the studies reported pre- and post-operative PROM scores. The mean weighted modified Harris Hip Score (mHHS) improved by 24.4% after hip arthroscopy (74.2 to 92.3). The mean weighted Nonarthritic Hip Score (NAHS) improved by 13.8% after hip arthroscopy (85.3 to 97.1) whilst the Hip Outcome Score sports (HOS-Sports) improved by 72.9% (47.2 to 81.6) and the Hip Outcome Score Activities of Daily Living (HOS ADL) improved by 24.8% (72.6 to 90.6). Table 3 shows the statistically significant improvements in all but one of the PROM scores in these studies (Table 4 + Fig. 2).

**Table 4:** Patient-reported outcome measures of the included studies

	Pre-Op	Post-Op	% improvement
mHHS	74.206	92.34717	24.4%
NAHS	85.3	97.1	13.8%
HOS-SPORT	47.2	81.62	72.9%
ADL HOS	72.56	90.584	24.8%
SF-12 (physical)	44	51	15.9%
SF-12 (mental)	49	54	10.2%
VAS	7.4	2.3	− 68.9%

### Return to play and follow-up

Return to play was reported in all 22 studies; however, only thirteen papers defined return to play as competing at the same competitive level as prior to surgery. Overall, the average return to play was 93.9% (95% CI: 90.5, 96.6) (I^2^ = 67.2%, *P* < 0.0001). The average return to play at the same competitive level was 94.9% (95% CI: 89.4, 98.4) (I^2^ = 75.1%, *P* < 0.0002). The average time to return to play was reported in 14 papers with a mean time of 6.8 ± 2.1 months.

**Fig. 3:**
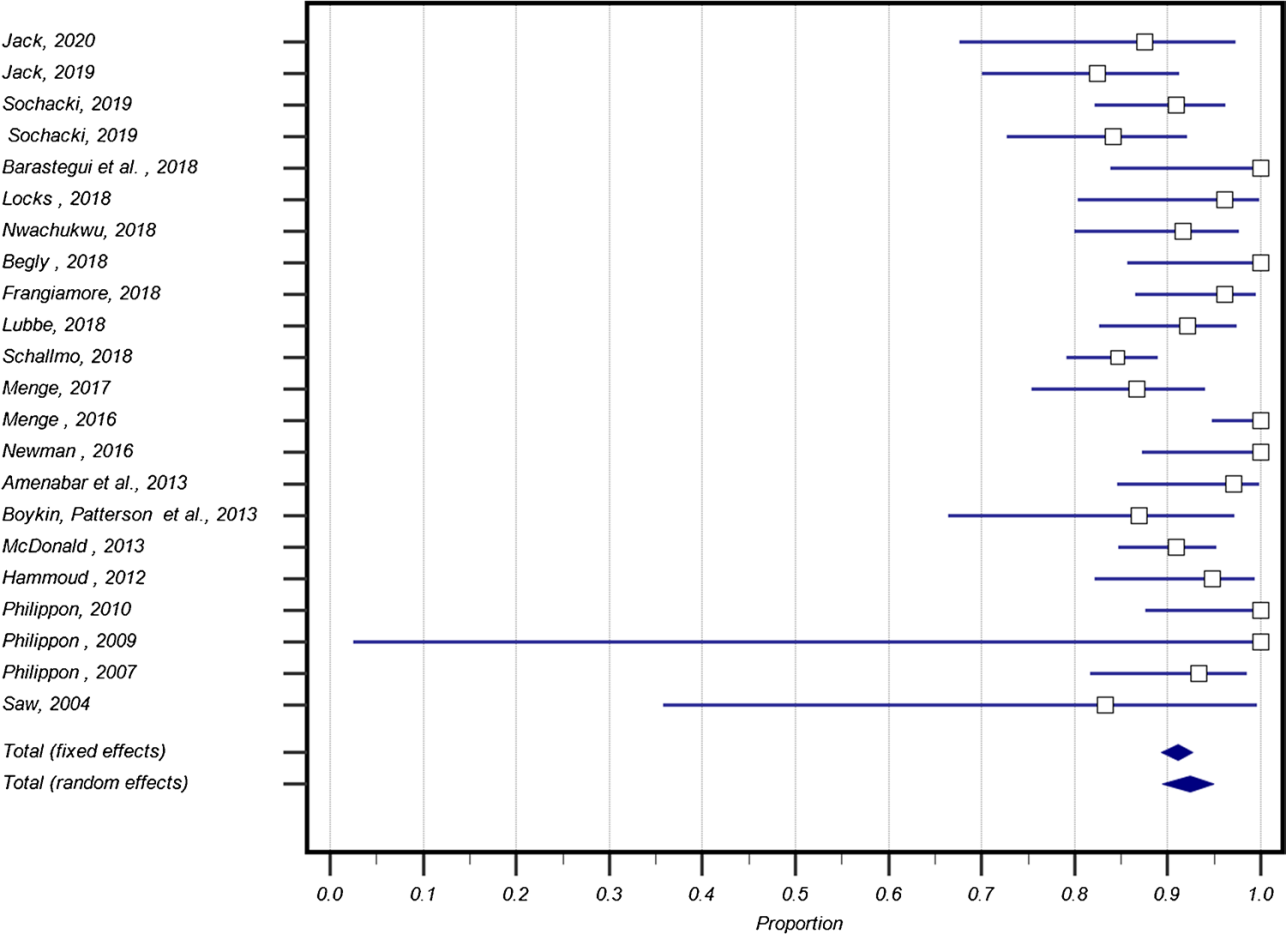
Individual study proportions and pooled estimate rates of return to sport

Nine papers reported that 22.8 ± 19.0% of patients had retired at the end of the follow-up period, which was 26.8 ± 19.3 months (reported in seven papers). Seventeen papers also reported on the average career length of the athletes post-arthroscopy up until the end of the follow-up period, eight of these were reported as years played and the other nine were seasons played, averaging at 3.6 ± 1.1 years and 3.2 ± 0.6 seasons respectively (Fig. 3 and Table 5).

**Table 5:** Career lengths and return to play of athletes

Author	Year	Patients (n)	Mean follow-up time (months)	RTP (% of players)	RTP time (months)	% retired at the end of last follow-up	% patients that required further intervention	Av. career length after hip arthroscopy at end of follow-up
Jack et al	2020	23	–	86.9*	5.7	–	–	4.4 seasons
Jack et al	2019	50	–	82.5*	8.3	4.0	–	3.3 years
Sochacki et al	2019	71	–	90.9*	6.8	15.6	–	3.3 years
Sochacki et al	2019	55	–	84.1*	6.7	20.6	5.4	3.5 years
Barastegui et al. [5]	2018	21	45.4	100*	10.8	47.6	–	2.1 seasons
Begly et al. [7]	2018	18	–	100*	7.1	–	–	4 seasons
Frangiamore et al. [13]	2018	44	–	95.5*	–	–	–	3.6 seasons
Locks et al. [22]	2018	24	–	96*	9.2	66.7	4.2	4.3 years
Lubbe et al	2018	64	–	92.6*	–	7.4	–	3.1 seasons
Nwachukwu et al. [36]	2018	40	–	92.5*	6	–	–	3.1 seasons
Schallmo et al. [46]	2018	180	–	84.6^*	6.9	–	–	2.7 years
Menge et al. [29]	2017	51	–	86.3	–	–	8.3	3.2 seasons
Menge et al. [30]	2016	60	–	100*	–	33.3	–	5.9 years
Newman et al. [35]	2016	20	–	100	4.7	–	10	5.72 years
Amenabar et al. [3]	2013	26	49.3	96	–	38	19.2	4.38 years
Boykin, et al. [9]	2013	21	41.4	85.7	–	–	19	3.6 seasons
McDonald et al. [27]	2013	120	–	90.8	–	–	–	2.9 seasons
Hammoud et al. [15]	2012	38	–	94.7	5.9	–	–	–
Philippon et al. [43]	2010	28	24	100	3.8	–	7.1	–
Philippon et al. [41]	2009	1	-	100	3.7	–	–	–
Philippon et al. [40]	2007	45	19.2	93.3*	–	22.2	11.1	–
Saw et al. [45]	2004	6	–	83.3	2.7	–	16.7	–

*These papers specified their RTP time as the time until the same level of competition as pre-surgery.

^This is the % of hips that returned to play since the paper did not report the % RTP of athletes.

Nine papers reported on the requirement for further surgery, with an average of 9.6% (95% CI: 5.2, 15.2) (I^2^ = 54.3%, *P* < 0.03) of patients requiring future intervention.

### Quality of the studies

All but four of the included studies were case series, with the exceptions being a case study, two cohort studies and a descriptive epidemiology study. The majority of these were level IV evidence. The papers ranged from serious to low risk of bias via ROBINS-I scoring, and MINORS scoring showed all but two of the included studies to be at high risk of bias. These are summarized in Table 1.

## Discussion

This systematic review evaluated 22 clinical studies with a total of 1006 patients and 1146 hips to determine the average RTP in the elite athletic population, as well as RTP time, career lengths and PROM scores. All studies reported a percentage of their athletic population that returned to play; however, only 13 papers (59.0%) reported a return to play at the same pre-operative competitive level. The overall RTP was 93.9% (95% CI: 90.5, 96.6) (I^2^ = 67.2%, *P* < 0.0001) and 94.9% (95% CI: 89.4, 98.4) (I^2^ = 75.1%, P < 0.0002) at the same competitive level, at an average time of 6.8 ± 2.1 months post-operative. This demonstrates a very high rate and speed of return to play in elite athletes. In contrast, O’Connor et al. [37] conducted a systematic review of return to sports in all levels of athletes after hip arthroscopy and found that the return to play rate was 84.6% (95% CI: 80.4%–88.8%; *P* = 0.008) with a mean time to RTP of 7.4 months (95% CI: 6.1–8.8). They found that the rate of return to sports within the recreational athletes in their cohort ranged from 66.7 to 84.0%. In another paper, Minkara et al. [31] systematically reviewed the outcomes of arthroscopic surgery for FAI and found that 87.7% of patients returned to sports after surgery. A higher RTP in elite athletes compared to recreational or amateur athletes is unsurprising given their high motivation for recovery and access to high-quality resources. Most elite athletes are professionals; therefore, their livelihoods, as well as their sporting achievements, depend on a good and fast outcome. When compared to open surgical dislocation, arthroscopy results in a faster rate of RTP for professional athletes; the technique is minimally invasive creating a lower complication rate and a faster return to activity [8]. Career lengths after arthroscopy were reported in 17 papers to be 3.2 ± 0.6 seasons and 3.6 ± 1.1 years. This data would be more useful if reported uniformly as years post-operation due to the variability of season lengths between sports. Menge et al. [29] found that 92% of their cohort of NFL players had a minimum total career length of three years, which equals the average career length of players within the NFL.

There were a variety of rehabilitation protocols throughout the papers, ranging from no specific restricted weight-bearing period [3] to four weeks of limited weight-bearing (8 weeks if microfracture) [40]. Despite many papers reporting longer rehabilitation times for microfracture, often eight weeks of limited weight-bearing, one paper found that elite athletes undergoing microfracture had no statistically significant difference in the rate of return to play [27]. Currently, rehabilitation protocols often depend on informal expert opinions rather than getting their basis from evidence, which at best is itself limited mostly to level IV evidence [37]. There needs to be more high-level evidence for rehabilitation protocols after hip arthroscopy, ideally randomized controlled trials looking at which protocols are most appropriate for athletes.

There was a lack of inclusion of standardized PROM scoring systems. Many of the studies did not include any PROMs, and for the few that did, there does not appear to be one scoring system that is favored against all of the others for a population of elite athletes. Many of the hip outcome scores were designed for activities of daily living and have limited application to sports activities, with the exception of HOS-Sports. We recommend that a consensus is needed on the most appropriate PROMs score for elite athletes, preferably one focused on sports-specific outcomes rather than activities of daily living, such as HOS-Sports, and reporting of these PROMs needs to be consistent across future studies.

A variety of sports were represented in the papers; however, the majority of sports included were the four major American sports (football, hockey, baseball and basketball), which may represent a bias in the data available. These sports have online databases, which allow for easy follow-up and retrospective analysis of return to sports and career length outcomes.

There needs to be a more standardized definition of “elite level”; it is often interchangeably with “competitive” or “professional”. Some papers seem to include high school, collegiate and professional athletes in an “elite”, “competitive” or “high-level” cohort [10, 47], whereas other papers defined elite athletes solely as professional athletes [27]. Even the definition of professional athletes is not definitive with Malviya et al. [25] defining anyone who played sports for a local club as a professional athlete. Swann et al. [52] found that there were eight different definitions of elite athletes within the literature, ranging from regional-level athletes with as little as two year experience in the sports to Olympic champions. They proposed a system for classification of elite status into four levels (based on answers to five variables): semi-elite (below the top standard of their sports), competitive elite (compete at the highest standard without success at that level), successful elite (compete at the highest standard with some success) and world-class elite (continued success over a prolonged period at the highest level). In order for future literature to allow for more specific definitions, we suggest more detail is needed in descriptions of athletic participation, especially the exact level of the league that the athletes compete in, their level of success and the length of time that they have competed at that level, which would allow for the application of Swann et al.’s (2015) classification system [52]. We defined “elite” as professional level or representation of their country because most of the literature seems to group athletes into high school, collegiate, professional or Olympic. Some papers included professional athletes in their cohorts; however, the data was impossible to extricate from the included high school and collegiate athletes. Whilst we recognize that some collegiate level athletes or even a few in high school may be playing at an elite level, there is a wide spectrum of level, which is difficult to regulate.

### Limitations

Despite following the established systematic guidelines, we recognize that this study has several limitations. Firstly, the majority of papers included in this systematic review are level IV evidence (i.e. case series). There is a lack of higher quality literature in the field, particularly on elite-level athletes as demonstrated here. Randomized or blinded studies may be particularly difficult to elicit on the elite athletic population because of the high pressure for each individual athlete to make a rapid return to sports. Accordingly, the level of evidence that this systematic review represents is reduced; however, the aggregate data collated on the 1006 athletes included in this paper allows for more robust estimates of data than that of any single study.

Secondly, there is a distinct lack of data on female elite athletes. Many of the included studies are on athletes exclusively in professional male leagues so there is a lack of data on female athletes. Previous studies have suggested that females in general have poorer outcomes than males for arthroscopic treatment of FAI [33], suggesting that the outcomes from male elite athletes undergoing hip arthroscopy may not be generalizable to the female elite athletic population. There was also variation in the inclusion criteria for the included studies; some papers only included patients who had undergone surgery for FAI, whereas others included all patients that had undergone hip arthroscopy and some included patients who had undergone specific procedures.

Although many papers reported average career lengths post-surgery, inherently this data is inaccurate because, in many cases, we cannot be clear on whether athletes’ careers ended due to reasons relating to their hip problems or from extrinsic factors. There is also a skew in the data because some athletes who are still competing at the end of the follow-up period may continue their athletic careers for many years past this point in time, likely creating an underestimate of career length.

Lastly, one of the major limitations faced when constructing this systematic review was the lack of a uniform definition of “elite”, as discussed previously. Lots of data had to be excluded due to its grouping with other athlete levels such as high school and collegiate. There is no individual breakdown of the athletes within the literature, which makes it impossible to extract this data. A consistent and universally agreed definition of “elite” needs to be utilized in the future to increase the quality of the literature and any conclusions made from it.

## Conclusion

This systematic review suggests that arthroscopic procedures in the hip (osteoplasty, chondroplasty, labral repair and/or debridement, capsulotomy, capsulorrhaphy or any soft tissue procedure) have good outcomes with 93.9% of athletes returning to play, often at the same level of competition as prior to the intervention. There is also a low rate of further interventions and good career lengths post-surgery, which suggest good future prognoses. Clearer reporting of elite athletes’ demographics is now needed for better classification of athletes into elite and non-elite categories within the literature. There is currently a large variation in rehabilitation methods; higher levels of evidence are required here to establish best practice guidelines for surgeons and physiotherapists.

## Data Availability

Yes, no supplementary data.

## References

[1] Agricola R, Heijboer MP, Bierma-Zeinstra SM, Verhaar JA, Weinans H, Waarsing JH (2013). Cam impingement causes osteoarthritis of the hip: a nationwide prospective cohort study (CHECK). Ann Rheum Dis.

[2] Alshameeri Z, Khanduja V (2014). The effect of femoro-acetabular impingement on the kinematics and kinetics of the hip joint. Int Orthop.

[3] Amenabar T, O’Donnell J (2013). Return to sport in Australian football league footballers after hip arthroscopy and midterm outcome. Arthroscopy.

[4] Anderson K, Strickland SM, Warren R (2001). Hip and groin injuries in athletes. Am J Sports Med.

[5] Barastegui D, Seijas R, Alvarez-Diaz P, Rivera E, Alentorn-Geli E, Steinbacher G (2018). Assessing long-term return to play after hip arthroscopy in football players evaluating risk factors for good prognosis. Knee Surg Sports Traumatol Arthrosc.

[6] Beck M, Kalhor M, Leunig M, Ganz R (2005). Hip morphology influences the pattern of damage to the acetabular cartilage: femoroacetabular impingement as a cause of early osteoarthritis of the hip. J Bone Joint Surg Br.

[7] Begly JP, Buckley PS, Utsunomiya H, Briggs KK, Philippon MJ (2018). Femoroacetabular impingement in professional basketball players: return to play, career length, and performance after hip arthroscopy. Am J Sports Med.

[8] Botser IB, Smith TW, Nasser R, Domb BG (2011). Open surgical dislocation versus arthroscopy for femoroacetabular impingement: a comparison of clinical outcomes. Arthroscopy.

[9] Boykin RE, Patterson D, Briggs KK, Dee A, Philippon MJ (2013). Results of arthroscopic labral reconstruction of the hip in elite athletes. Am J Sports Med.

[10] Degen RM, Fields KG, Wentzel CS, Bartscherer B, Ranawat AS, Coleman SH (2016). Return-to-play rates following arthroscopic treatment of femoroacetabular impingement in competitive baseball players. Phys Sportsmed.

[11] Drakos MC, Domb B, Starkey C, Callahan L, Allen AA (2010). Injury in the national basketball association: a 17-year overview. Sports Health.

[12] Feeley BT, Kennelly S, Barnes RP, Muller MS, Kelly BT, Rodeo SA (2008). Epidemiology of National Football League training camp injuries from 1998 to 2007. Am J Sports Med.

[13] Frangiamore SJ, Mannava S, Briggs KK, McNamara S, Philippon MJ (2018). Career length and performance among professional baseball players returning to play after hip arthroscopy. Am J Sports Med.

[14] Frangiamore SJ, Mannava S, Briggs KK, McNamara S, Philippon MJJTAJoSM (2018) Career length and performance among professional baseball players returning to play after hip arthroscopy. 0363546518775420 10.1177/0363546518775420 29799269

[15] Hammoud S, Bedi A, Magennis E, Meyers WC, Kelly BT (2012). High incidence of athletic pubalgia symptoms in professional athletes with symptomatic femoroacetabular impingement. Arthroscopy.

[16] Jack RA, Sochacki KR, Hirase T, Vickery J, McCulloch PC, Lintner DM (2020). Performance and return to sport after hip arthroscopy in the National Basketball Association. Arthroscopy.

[17] Jack RA, Sochacki KR, Hirase T, Vickery J, McCulloch PC, Lintner DM (2019). Performance and return to sport after hip arthroscopic surgery in Major League Baseball Players. Orthop J Sports Med.

[18] Jackson TJ, Starkey C, McElhiney D, Domb BG (2013). Epidemiology of hip injuries in the National Basketball Association: a 24-year overview. Orthop J Sports Med.

[19] Khanduja V, Villar RN (2007). The arthroscopic management of femoroacetabular impingement. Knee Surg Sports Traumatol Arthrosc.

[20] Kowalczuk M, Yeung M, Simunovic N, Ayeni OR (2015). Does femoroacetabular impingement contribute to the development of hip osteoarthritis? A systematic review. Sports Med Arthrosc Rev.

[21] Kujala UM, Kaprio J, Sarna S (1994). Osteoarthritis of weight bearing joints of lower limbs in former elite male athletes. BMJ.

[22] Locks R, Utsunomiya H, Briggs KK, McNamara S, Chahla J, Philippon MJ (2018). Return to play after hip arthroscopic surgery for femoroacetabular impingement in professional soccer players. Am J Sports Med.

[23] Lubbe RJ, Freshman RD, Singh G, Katchko KM, Schneider AD, Sharma S, et al. (2018) Performance outcomes and return-to-sport rate of National Hockey League athletes vary after common orthopedic surgical procedures. Clin J Sport Med. 10.1097/JSM.0000000000000696 10.1097/JSM.0000000000000696 30439727

[24] Mai HT, Alvarez AP, Freshman RD, Chun DS, Minhas SV, Patel AA (2016). The NFL Orthopaedic Surgery Outcomes Database (NO-SOD): the effect of common orthopaedic procedures on football careers. Am J Sports Med.

[25] Malviya A, Paliobeis CP, Villar RN (2013). Do professional athletes perform better than recreational athletes after arthroscopy for femoroacetabular impingement?. Clin Orthop Relat Res.

[26] McDonald JE, Herzog MM, Philippon MJ (2014). Performance outcomes in professional hockey players following arthroscopic treatment of FAI and microfracture of the hip. Knee Surg Sports Traumatol Arthrosc.

[27] McDonald JE, Herzog MM, Philippon MJ (2013). Return to play after hip arthroscopy with microfracture in elite athletes. Arthroscopy.

[28] McKay CD, Tufts RJ, Shaffer B, Meeuwisse WH (2014). The epidemiology of professional ice hockey injuries: a prospective report of six NHL seasons. Br J Sports Med.

[29] Menge TJ, Bhatia S, McNamara SC, Briggs KK, Philippon MJ (2017). Femoroacetabular impingement in professional football players: return to play and predictors of career length after hip arthroscopy. Am J Sports Med.

[30] Menge TJ, Briggs KK, Philippon MJ (2016). Predictors of length of career after hip arthroscopy for femoroacetabular impingement in professional hockey players. Am J Sports Med.

[31] Minkara AA, Westermann RW, Rosneck J, Lynch TS (2019). Systematic review and meta-analysis of outcomes after hip arthroscopy in femoroacetabular impingement. Am J Sports Med.

[32] Munegato D, Bigoni M, Gridavilla G, Olmi S, Cesana G, Zatti G (2015). Sports hernia and femoroacetabular impingement in athletes: a systematic review. World J Clin Cases.

[33] Mygind-Klavsen B, Lund B, Nielsen TG, Maagaard N, Kraemer O, Holmich P (2019). Danish Hip Arthroscopy Registry: predictors of outcome in patients with femoroacetabular impingement (FAI). Knee Surg Sports Traumatol Arthrosc.

[34] Nepple JJ, Vigdorchik JM, Clohisy JC (2015). What is the association between sports participation and the development of proximal femoral cam deformity? A systematic review and meta-analysis. Am J Sports Med.

[35] Newman JT, Saroki AJ, Briggs KK, Philippon MJ (2016). Return to elite level of play and performance in professional golfers after arthroscopic hip surgery. Orthop J Sports Med.

[36] Nwachukwu BU, Bedi A, Premkumar A, Draovitch P, Kelly BT (2018). Characteristics and outcomes of arthroscopic femoroacetabular impingement surgery in the National Football League. Am J Sports Med.

[37] O’Connor M, Minkara AA, Westermann RW, Rosneck J, Lynch TS (2018). Return to play after hip arthroscopy: a systematic review and meta-analysis. Am J Sports Med.

[38] Obremskey WT, Pappas N, Attallah-Wasif E, Tornetta P, Bhandari M (2005). Level of evidence in orthopaedic journals. J Bone Joint Surg Am.

[39] Packer JD, Safran MR (2015). The etiology of primary femoroacetabular impingement: genetics or acquired deformity?. J Hip Preserv Surg.

[40] Philippon M, Schenker M, Briggs K, Kuppersmith D (2007). Femoroacetabular impingement in 45 professional athletes: associated pathologies and return to sport following arthroscopic decompression. Knee Surg Sports Traumatol Arthrosc.

[41] Philippon MJ, Christensen JC, Wahoff MS (2009). Rehabilitation after arthroscopic repair of intra-articular disorders of the hip in a professional football athlete. J Sport Rehabil.

[42] Philippon MJ (2005). Schenker MLJOTiO. Athletic hip injuries and capsular laxity.

[43] Philippon MJ, Weiss DR, Kuppersmith DA, Briggs KK, Hay CJ (2010). Arthroscopic labral repair and treatment of femoroacetabular impingement in professional hockey players. Am J Sports Med.

[44] Posner M, Cameron KL, Wolf JM, Belmont PJ, Owens BD (2011). Epidemiology of Major League Baseball injuries. Am J Sports Med.

[45] Saw T, Villar R (2004). Footballer’s hip a report of six cases. J Bone Joint Surg Br.

[46] Schallmo MS, Fitzpatrick TH, Yancey HB, Marquez-Lara A, Luo TD, Stubbs AJ (2018). Return-to-play and performance outcomes of professional athletes in North America after hip arthroscopy from 1999 to 2016. Am J Sports Med.

[47] Shibata KR, Matsuda S, Safran MR (2017). Arthroscopic hip surgery in the elite athlete: comparison of female and male competitive athletes. Am J Sports Med.

[48] Shindle MK, Voos JE, Heyworth BE, Mintz DN, Moya LE, Buly RL (2007). Hip arthroscopy in the athletic patient: current techniques and spectrum of disease. J Bone Joint Surg Am.

[49] Siebenrock KA, Behning A, Mamisch TC, Schwab JM (2013). Growth plate alteration precedes cam-type deformity in elite basketball players. Clin Orthop Relat Res.

[50] Sochacki KR, Jack RA, Hirase T, Vickery J, McCulloch PC, Lintner DM (2019). Performance and return to sport after femoroacetabular impingement surgery in National Football League players. Orthopedics.

[51] Sochacki KR, Jack RA, Hirase T, Vickery J, Harris JD (2019). Performance and return to sport after hip arthroscopy for femoracetabular impingement syndrome in National Hockey League players. Journal of Hip Preservation Surgery.

[52] Swann C, Moran A, Piggott D (2015). Defining elite athletes: issues in the study of expert performance in sport psychology. Psychol Sport Exerc.

[53] Swartz MK (2011). The PRISMA statement: a guideline for systematic reviews and meta-analyses. J Pediatr Health Care.

[54] Takeyama A, Naito M, Shiramizu K, Kiyama T (2009). Prevalence of femoroacetabular impingement in Asian patients with osteoarthritis of the hip. Int Orthop.

[55] Tveit M, Rosengren BE, Nilsson JA, Karlsson MK (2012). Former male elite athletes have a higher prevalence of osteoarthritis and arthroplasty in the hip and knee than expected. Am J Sports Med.

[56] Vingard E, Alfredsson L, Goldie I, Hogstedt C (1993). Sports and osteoarthrosis of the hip. An epidemiologic study. Am J Sports Med.

